# Automated three-dimensional left atrial analysis on computed tomography angiography: reproducibility and workflow efficiency of eight clinically relevant metrics using a deep learning pipeline

**DOI:** 10.3389/fbioe.2025.1697542

**Published:** 2026-01-15

**Authors:** Youqi Fan, Jian Ye, Xiaoya Wang, Liuguang Song, Yaping Wang

**Affiliations:** 1 Department of Cardiology, The Second Affiliated Hospital, Zhejiang University School of Medicine, Hangzhou, China; 2 State Key Laboratory of Transvascular Implantation Devices, Hangzhou, China; 3 Heart Regeneration and Repair Key Laboratory of Zhejiang province, Hangzhou, China

**Keywords:** computed tomography angiography, deep learning, left atrial appendage, left atrium, pulmonary veins

## Abstract

**Background:**

Accurate and reproducible quantification of LA anatomy from CTA is essential for ablation, LAAC, and structural interventions, yet manual measurements are time-consuming and prone to inter-observer variability.

**Objectives:**

To validate a deep learning–based CTA pipeline for automated quantification of eight clinically relevant LA metrics, and to assess its agreement, repeatability, and efficiency compared with expert measurements.

**Methods:**

In this retrospective study, 407 patients were included and divided into training (n = 270), validation (n = 87), and clinical evaluation (n = 50) cohorts. A MedNeXt-based model performed multi-structure segmentation, and a geometry-driven framework computed eight metrics: LA volume, LAA volume, AP/ML/SI diameters, left/right PV ostial size, left/right PV inter-ostial angles, and LAA ostial size. Automated outputs were compared with expert annotations using intraclass correlation coefficients (ICCs) and Bland–Altman analysis (primary endpoints: LA volume and diameters). Workflow efficiency and usability (Likert scale, 1–5) were also assessed by electrophysiology/structural experts.

**Results:**

Automated measurements demonstrated excellent agreement with experts for primary endpoints (LA volume ICC = 0.999; AP/ML/SI diameter ICCs = 0.972–0.985), with minimal bias and narrow limits of agreement. Agreement for other metrics was good to excellent (typical ICCs ≥0.84). Analysis time was reduced from 15.3 ± 1.4 min to 0.5 ± 0.1 min per case (≈92% reduction; p < 0.001). Usability ratings were ≥4/5 in most cases, with 63%–76% classified as Grade A (fully usable without manual edits). Performance remained consistent across voxel-size strata.

**Conclusion:**

The proposed pipeline enables rapid, reproducible, and expert-level quantification of eight LA metrics on CTA, demonstrating technical feasibility for clinical integration pending prospective validation of procedural impact.

## 1 Introduction

Quantitative assessment of the LA, LAA, and PVs informs several clinical decisions. LA volume and linear diameters are associated with AF recurrence and thromboembolic risk (Tsang et al., 2001; Abhayaratna et al., 2006; Zhuang et al., 2012); PV ostial size and inter-ostial angles influence catheter access and lesion formation during AF ablation (Ho et al., 2001; Mansour et al., 2004; Kato et al., 2003); and LAA volume and ostial size guide device selection and safety for LAAC (Wang et al., 2010; Budge et al., 2008; Nucifora et al., 2011). Manual measurements on cardiac CTA are time-consuming and show inter-reader variability (Dewey et al., 2007; Mahnken et al., 2005; Dewey et al., 2006). We developed an automation-first, definition-transparent pipeline to reproduce eight routine metrics with expert-level agreement, and to report per-case success grades and runtime as indicators of workflow readiness. Rather than modeling clinical outcomes, we restrict outputs to metrics already supported by common practice (Calkins et al., 2017; January et al., 2019; Holmes et al., 2014), positioning the tool as a practical bridge from manual to automated pre-procedural planning.

## 2 Materials and methods

### 2.1 Study design and cohorts

This single-center study was approved by the Ethics Committee of the Second Affiliated Hospital of Zhejiang University School of Medicine (approval No. 2025–0777). Inclusion criteria were: age≥18 years; availability of cardiac CTA performed with a standardized protocol; and complete clinical and follow-up data. Exclusion criteria were: prior cardiac surgery; major structural abnormalities affecting LA anatomy; contraindication to iodinated contrast or renal insufficiency; and poor image quality due to motion or technical artifacts. The dataset was randomly partitioned without subject overlap into training (n = 270), validation (n = 87), and clinical evaluation (n = 50) cohorts (approximately 7:2:1).

### 2.2 Acquisition protocol

Cardiac CTA was acquired on a Philips iCT 256 scanner using prospective ECG gating at 70%–80% of the R-R interval. Iohexol 370 mg I/mL was injected via an antecubital vein at 0.7 mL/kg (maximum 70 mL) at 4 mL/s. Acquisition was triggered 15 s after contrast arrival in the descending aorta. Images were reconstructed with a cardiac-standard kernel (“Cb”). The typical in-plane pixel size was 0.5 mm and the native slice thickness 0.65 mm.

### 2.3 Annotation and reference measurements

Two board-certified radiologists (>10 years’ experience in cardiac CTA) independently performed manual segmentations and reference measurements in 3D Slicer (v5.6.0; RRID:SCR_005619) to establish the ground-truth dataset for training, validation, and testing. A calibration session on 20 pilot cases was conducted to harmonize measurement techniques; inter-observer ICC for LA volume was 0.97 (95% CI: 0.94–0.99) and for PV ostial diameters was 0.89–0.92. All results were reviewed by two quality-control experts; disagreements were resolved by a third adjudicator. Anatomical definitions followed the 2020 EHRA/HRS/APHRS/LAHRS expert consensus statement on LA anatomy and function (Hindricks et al., 2020). The measurement set comprised LA chamber dimensions, LA wall thickness, and spatial relations to adjacent structures.

### 2.4 Segmentation model

We developed a multi-structure segmentation network based on MedNeXt (Roy et al., 2023) for simultaneous delineation of the aorta, LA, LAA, and PV sleeves. The encoder comprises dense convolutional blocks with channel-attention gates; the decoder uses progressive up-sampling with skip connections and learned deconvolution filters. A multi-head self-attention bottleneck captures long-range context to enhance boundary delineation. MedNeXt was selected over alternatives for its superior performance in multi-structure cardiac segmentation, with validation-set DSC improvements of 1.2%–1.8% for LAA and PV structures.

Training data (n = 270) were resampled to an isotropic resolution of 0.5 mm and intensity-normalized. The input volume size was 112*192*192 voxels. The loss combined weighted Dice (w = 1.0), cross-entropy (w = 0.5), and topology-preserving terms (w = 0.3) to address class imbalance and complex anatomy. Post-processing included connected component analysis and morphological closing. Data augmentation included random rotation, scaling, mirroring, elastic deformation, intensity shifts, cropping, contrast adjustments, and Gaussian noise/blur.

Optimization used AdamW (initial learning rate 0.01, cosine decay schedule), batch size 4, and 500 epochs (250 iterations per epoch). Training was performed for 24 h on a single NVIDIA GeForce RTX 3090 GPU.

Environment. Experiments ran on Ubuntu 24.04 LTS with Python 3.12, PyTorch 2.4 (CUDA 12.4), and TensorRT 10.1 on hardware comprising two Intel Xeon Platinum 8375C CPUs and two NVIDIA GeForce RTX 3090 GPUs (24 GB each).

### 2.5 Geometry framework

Geometric analysis was performed on binary masks of the LA, LAA, and individual PV sleeves produced by the segmentation model. Prior to measurement, all label maps were resampled to isotropic 0.5-mm spacing to standardize distance and area calculations and to limit partial-volume effects. A basal reference plane was fitted to the mitral annulus, and orthogonal anatomical axes were established to approximate anteroposterior (AP), mediolateral (ML), and superoinferior (SI) directions for diameter computations. PV and LAA centerlines were extracted to localize ostia and define local orientations for plane placement.

Volumes. LA chamber volume and LAA volume were computed from their respective masks by voxel summation on the resampled grids. For LA volume, the LAA and PV sleeves were excluded at their ostial planes (defined below) to avoid overestimation; the LAA volume was integrated distal to the LAA ostial plane.

LA diameters (AP, ML, SI). Diameters were derived from orthogonal plane sweeps that emulate clinical caliper placement while avoiding bias from the LAA and PV sleeves. For AP and ML diameters, axial slices spanning the LA were traversed from inferior to superior; on each slice, the LA contour (with LAA/PV sleeves excluded) was identified and its anterior–posterior and left–right chord lengths computed. The maximum AP and ML chord lengths across slices were recorded as the AP and ML diameters. For the SI diameter, sagittal slices spanning the LA were traversed from left to right; the superior-inferior chord within the LA contour was computed per slice, and the maximum across slices taken as the SI diameter. This slice-wise maximum-chord strategy reproduces routine measurement practice, is robust to local irregularities, and confines measurements to the LA cavity.

PV ostium equivalent diameter (left and right). For each PV sleeve, an ostial plane at the LA–PV junction was determined using the masks alone. Along the PV centerline approaching the LA, orthogonal cross-sections were generated within ±5 mm of the junction; the cross-section with the minimal enclosed area that still intersected both labels was designated the ostium. From this cross-section, the equivalent circular diameter was reported. A single left-sided and right-sided PV ostial diameter was obtained by averaging the superior and inferior veins on each side, while vein-specific values were retained for quality control and supplementary analysis.

PV inter-ostial angles (left and right). Left-sided (superior vs. inferior) and right-sided (superior vs. inferior) inter-ostial angles were derived from local flow-oriented vectors at each ostium. For each PV, the centerline direction at the ostium was defined by the vector connecting two points sampled at±5 mm from the ostial plane along the centerline. The 3D angle between the superior and inferior vectors on a given side was then calculated. These angles are part of the primary metric set.

LAA ostium equivalent diameter. The LAA ostium was defined analogously to PV ostia. An area-minimizing cross-section orthogonal to the LAA centerline within ±5 mm of the LA–LAA junction was selected, and the equivalent circular diameter was reported.

To minimize sensitivity to resolution and partial-volume effects, all computations were performed on isotropically resampled masks; native voxel sizes and reconstruction kernels were recorded; and sensitivity analyses were prespecified by native voxel-size strata. Ostial plane selection was constrained to a narrow neighborhood (±5 mm) to prevent plane drift, and topology checks ensured single, anatomically plausible intersections at each junction. The same geometry framework was applied identically to automated segmentations and expert manual masks, ensuring that metric definitions and computations were independent of the segmentation source.

### 2.6 Usability and time-efficiency evaluation

To evaluate clinical usability, we compared the time required for automated quantification with manual measurements performed by junior and senior observers. For each patient, the framework simultaneously generated eight predefined metric categories: (1) LA volume; (2–4) AP/ML/SI diameters; (5) left and right PV ostial equivalent diameters; (6) left and right PV inter-ostial angles; (7) LAA volume; and (8) LAA ostial equivalent diameter. Manual measurements were performed in a blinded fashion using multiplanar reconstruction tools. Time was recorded from loading the CTA dataset to completion of all measurements (GPU: NVIDIA RTX 3090).

Usability grading was performed independently by two blinded experts (a senior electrophysiologist and a structural interventionalist, each with >10 years’ CTA planning experience) using structured criteria. Inter-rater agreement was assessed using Cohen’s kappa; disagreements were adjudicated by a third reviewer.

Output usability was categorized into three grades based on whether key categories of measurements could be reliably obtained:

Grade A: all three categories of measurements were successfully extracted, including (i) left atrial (LA) volume and diametric indices, (ii) pulmonary vein (PV) ostial angle and configuration, and (iii) left atrial appendage (LAA) volume and ostial plane.

Grade B: two categories were successfully derived, while the third required partial or complete manual correction.

Grade C: only one category could be reliably obtained, or severe errors necessitated full manual remeasurement.

This grading provides a clinically interpretable evaluation of framework performance, consistent with prior work in automated cardiac CTA analysis.

### 2.7 Statistical analysis

Continuous variables are summarized as mean ± standard deviation (SD) or median (interquartile range), as appropriate. Categorical variables are reported as counts and percentages. Segmentation performance was assessed using Dice similarity coefficient (DSC), surface Dice coefficient (SDC), and the 95th percentile Hausdorff distance (HD95). Agreement between automated and manual measurements was quantified using intraclass correlation coefficients (two-way mixed model, absolute agreement) and Bland-Altman analysis.

Time efficiency between automated and manual methods was compared using paired Student’s t-tests or Wilcoxon signed-rank tests, depending on distributional assumptions. Differences in categorical variables, including the Grade A/B/C usability classification, were assessed using chi-square or Fisher’s exact tests. Statistical analyses were conducted using Python (version 3.12.3), with libraries including numpy (version 1.26.0), pandas (version 2.1.4), scipy (version 1.11.2), and statsmodels (version 0.14.1). The evaluation cohort sample size (n = 50) was determined to achieve 95% CI widths ≤0.10 for ICC estimates ≥0.90, consistent with prior cardiac measurement validation studies. Two-sided p-values <0.05 were considered statistically significant; where possible, exact p values and 95% confidence intervals are reported alongside effect sizes.

## 3 Results

### 3.1 Study population

**FIGURE 1:**
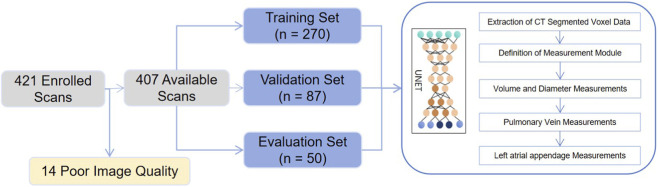
Flow chart. A workflow diagram showing 421 enrolled scans, with 14 excluded due to poor image quality. The remaining 407 scans are divided into training (270), validation (87), and evaluation (50) sets. These sets are utilized to train and evaluate a UNet model for measurements of CT segmented data, including volume, diameter, pulmonary veins, and the left atrial appendage.

As described in the Methods, Baseline and demographic characteristics of the enrolled subjects are presented in Table 1. Age and sex distributions were comparable across the training sets (n = 270), validation sets (n = 87), and clinical evaluation sets (n = 50) cohorts (mean age: 67.1 ± 9.5, 66.8 ± 8.7, and 67.3 ± 9.1 years, respectively; P = 0.073; male proportion: 60.4%, 62.1%, and 60.0%; P = 0.429). Among all baseline variables, only body-mass index (BMI) and the prevalence of coronary-artery disease exhibited statistically significant inter-cohort differences (BMI: 27.1 ± 3.6, 26.8 ± 3.9, and 28.5 ± 4.0 kg m-2, P = 0.028, one-way ANOVA; coronary-artery disease: 28.9% [78/270], 17.2% [15/87], and 40.0% [20/50], P = 0.005,chi-square test). The overall study workflow is depicted in Figure 1.

**TABLE 1:** Baseline characteristics.

Characteristic	Training set (n = 270)	Validation set (n = 87)	Test set (n = 50)	P value
Age, years	67.1 ± 9.5	66.8 ± 8.7	67.3 ± 9.1	0.073
Male	163 (60.4%)	54 (62.1%)	30 (60.0%)	0.429
Female	107 (39.6%)	33 (37.9%)	20 (40.0%)	0.994
Body mass index, kg/m2	27.1 ± 3.6	26.8 ± 3.9	28.5 ± 4.0	0.028
Smoking history	95 (35.2%)	20 (23.0%)	18 (36.0%)	0.083
Hypertension	142 (52.6%)	46 (52.9%)	28 (56.0%)	0.994
Diabetes mellitus	61 (22.6%)	20 (23.0%)	11 (22.0%)	0.906
Atrial fibrillation	85 (31.5%)	28 (32.2%)	17 (34.0%)	0.913
Left atrial enlargement	93 (34.4%)	30 (34.5%)	17 (34.0%)	0.992
Coronary artery disease	78 (28.9%)	15 (17.2%)	20 (40.0%)	0.005
Chronic kidney disease	34 (12.6%)	12 (13.8%)	5 (10.0%)	0.769
Hyperlipidemia	120 (44.4%)	35 (40.2%)	20 (40.0%)	0.776

Data are presented as mean ± standard deviation or n (%).

### 3.2 Segmentation performance

**FIGURE 2:**
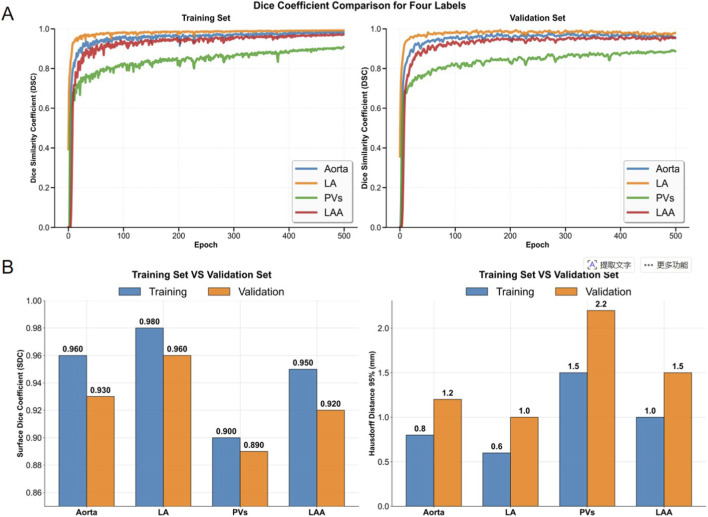
Performance of the MedNeXt-based network for multi-structure cardiac segmentation. **(A)** Dice coefficient curves during training and validation for the aorta, LA, PV sleeves, and LAA, demonstrating rapid convergence and stable high accuracy. **(B)** Comparison of training versus validation sets for surface Dice coefficient (SDC, left) and 95th percentile Hausdorff distance (HD95, right), confirming consistent generalization across datasets with minimal performance drop between training and validation cohorts.

The MedNeXt-based network achieved high-fidelity segmentation across the LA cavity, LAA, PV sleeves, and thoracic aorta (Figure 2). Validation metrics demonstrated robust performance: LA (DSC 0.984, SDC 0.928, HD95 6.5 mm), PV sleeves (DSC 0.950, SDC 0.881, HD95 5.5 mm), thoracic aorta (DSC 0.971, SDC 0.940, HD95 6.0 mm), and LAA (DSC 0.944, SDC 0.913, HD95 7.0 mm). The consistently high surface Dice coefficients (>0.88) and low HD95 distances (<7 mm) highlight precise boundary delineation, particularly at the anatomically complex LA–LAA and LA–PV junctions, thereby facilitating reliable ostial plane definition for downstream quantitative analyses.

### 3.3 Usability and time-efficiency

The proposed framework successfully generated all eight predefined quantitative metrics in the majority of cases. In the validation cohort, grading outcomes were as follows: Grade A in 55 of 87 cases (63.2%), Grade B in 30 cases (34.5%), and Grade C in 2 cases (2.3%). In the independent clinical evaluation cohort, 38 of 50 cases (76.0%) were classified as Grade A, 11 cases (22.0%) as Grade B, and 1 case (2.0%) as Grade C (Table 2). Grade B cases generally required minor manual refinements of pulmonary venous ostial contours, whereas Grade C cases were predominantly attributable to pronounced motion artifacts or highly atypical cardiac anatomy.

**TABLE 2:** Distribution of grading outcomes in the validation (n = 87) and clinical evaluation (n = 50) cohorts.

Grade	Validation set (n = 87)	Evaluation set (n = 50)	P
A	55	37	0.257
B	30	12	0.249
C	2	1	1

**FIGURE 3:**
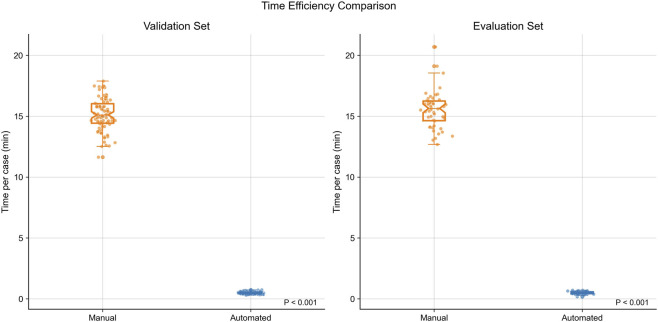
Comparison of time requirements for manual versus automated quantification across the validation set (left) and clinical evaluation set (right). Automated analysis reduced per-case time from a median of approximately 15 min to less than 1 min, yielding a >90% relative reduction (p < 0.001 for both cohorts).

Automated quantification required only 0.5 ± 0.1 min per case, in contrast to 15.3 ± 1.4 min for manual analysis of the same eight categories (two-sided p < 0.001; Figure 3). The median time reduction per case was 15.0 min [IQR 13.5–16.1], corresponding to a relative efficiency gain of 91.7% (Wilcoxon signed-rank test, p < 0.001). Inter-rater agreement for usability grading was substantial (Cohen’s kappa = 0.82 [95% CI: 0.74–0.89]).

### 3.4 Agreement, distribution, and sensitivity analyses

Automated measurements demonstrated excellent concordance with expert annotations across all evaluated metrics in both cohorts. In the validation cohort (n = 87), intraclass correlation coefficients (ICCs; two-way mixed, absolute agreement) were 0.999 (95% CI: 0.999–1.000) for LA volume and 0.950 (0.925–0.967) for LAA volume, with Bland–Altman biases of 0.866 ± 5.221 mL (limits of agreement [LOA] −9.59–11.32 mL) and −0.566 ± 4.537 mL (LOA −9.64 to 8.51 mL), respectively. In the independent evaluation cohort (n = 50), ICCs remained similarly high (0.999 [0.999–1.000] for LA volume; 0.916 [0.858–0.952] for LAA volume), with small biases of −0.421 ± 4.931 mL (LOA −10.28 to 9.44 mL) and −1.759 ± 5.116 mL (LOA −11.93 to 8.41 mL).

For LA diameters (AP, ML, SI), ICCs in the validation cohort were 0.980 (0.970–0.987), 0.985 (0.977–0.990), and 0.985 (0.977–0.990), with negligible biases (−0.013 ± 2.665 mm, 0.138 ± 2.345 mm, and 0.435 ± 2.087 mm). The evaluation cohort showed comparably high ICCs (0.978 [0.961–0.987], 0.985 [0.974–0.992], and 0.972 [0.952–0.984]) with minimal biases (−0.565 ± 2.715 mm, −0.536 ± 2.685 mm, and 0.072 ± 3.034 mm). No proportional bias was detected for any of the diameter measurements (all p > 0.05).

Pulmonary venous (PV) ostial areas also exhibited strong reproducibility. In the validation cohort, left superior PV equivalent diameter showed an ICC of 0.845 (0.772–0.896) with a bias of 0.115 ± 1.450 mm^2^, while the evaluation cohort demonstrated an ICC of 0.844 (0.742–0.908) with a bias of −0.120 ± 1.480 mm^2^. Right PV ostial areas showed similar ICCs (0.836–0.818) with biases <1.1 mm^2^. Equivalent diameter metrics and inter-ostial angle measurements (typical ICC ≈0.82–0.85) demonstrated moderate-to-strong agreement, with LOAs consistently within clinically acceptable thresholds (<10% of mean values).

**FIGURE 4:**
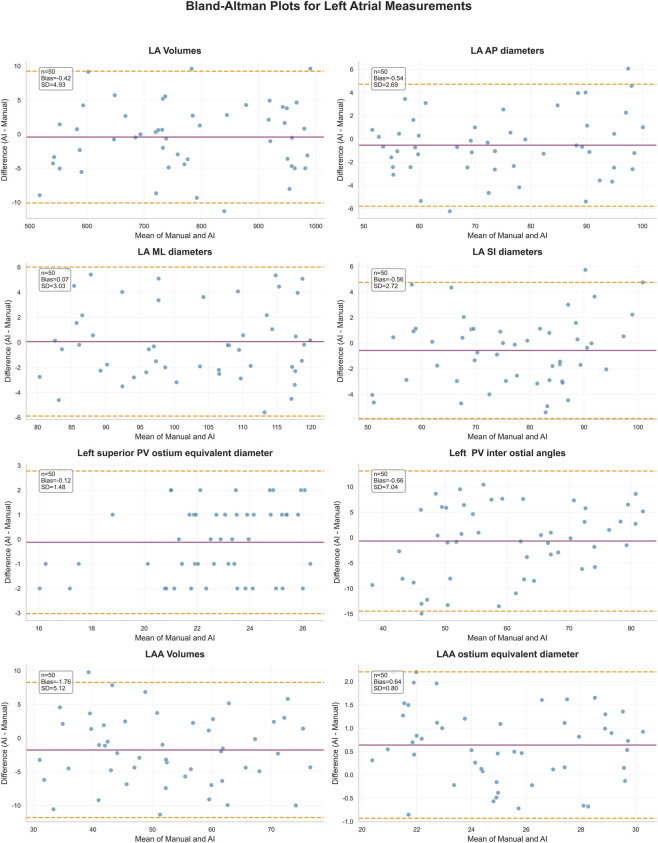
Representative Bland–Altman plots showing agreement between automated and expert-derived measurements for selected metrics from the predefined eight-category framework. Illustrated indices include LA volume, LA diameters (AP, ML, SI), left superior PV ostial equivalent diameter, left PV inter-ostial angle, LAA volume, and LAA ostial equivalent diameter. Plots demonstrate minimal systematic bias and narrow limits of agreement, confirming excellent reproducibility of automated analysis.

For the LAA ostium, ICCs were 0.942 (0.912–0.961) in the validation cohort and 0.942 (0.900–0.966) in the evaluation cohort, with very small biases for both area and equivalent diameter. Representative Bland–Altman plots (Figure 4) further confirmed minimal systematic bias and narrow LOAs across all major metrics. Full comparative statistics for both cohorts are summarized in Table 3.

**TABLE 3:** Agreement between automated measurements and expert annotations in the validation set (n = 87) and evaluation set (n = 50).

Measure	Validation set (n = 87)			Evaluation set (n = 50)		
	Bias ±SD	ICC [95% CI]	P	Bias ±SD	ICC [95% CI]	P
LA volumes	0.87 ± 5.22	0.99 [0.99, 1.00]	0.125	−0.42 ± 4.93	0.99 [0.99, 1.00]	0.548
LA AP diameters	0.14 ± 2.35	0.99 [0.98, 0.99]	0.584	−0.54 ± 2.69	0.99 [0.97, 0.99]	0.164
LA ML diameters	0.44 ± 2.09	0.99 [0.98, 0.99]	0.055	0.07 ± 3.03	0.97 [0.95, 0.98]	0.868
LA SI diameters	−0.01 ± 2.66	0.98 [0.97, 0.99]	0.965	−0.57 ± 2.71	0.98 [0.96, 0.99]	0.147
Left superior PV ostium equivalent diameter	0.12 ± 1.45	0.85 [0.77, 0.90]	0.461	−0.12 ± 1.48	0.84 [0.73, 0.90]	0.569
Left inferior PV ostium equivalent diameter	0.06 ± 1.47	0.85 [0.78, 0.90]	0.715	−0.12 ± 1.53	0.83 [0.73, 0.90]	0.582
Right superior PV ostium equivalent diameter	0.03 ± 1.74	0.88 [0.83, 0.92]	0.828	0.36 ± 1.30	0.90 [0.82, 0.94]	0.057
Right inferior PV ostium equivalent diameter	0.07 ± 1.58	0.85 [0.77, 0.90]	0.685	0.16 ± 1.43	0.84 [0.74, 0.91]	0.434
Left PV inter ostial angles	0.22 ± 6.93	0.85 [0.77, 0.90]	0.770	−0.66 ± 7.04	0.84 [0.74, 0.90]	0.513
Right PV inter ostial angles	1.03 ± 7.21	0.84 [0.76, 0.89]	0.188	0.56 ± 6.89	0.82 [0.70, 0.89]	0.566
LAA volumes	−0.57 ± 4.53	0.95 [0.93, 0.97]	0.248	−1.76 ± 5.12	0.92 [0.86, 0.95]	0.019
LAA ostium equivalent diameters	0.66 ± 0.76	0.94 [0.91, 0.96]	<0.005	0.64 ± 0.80	0.94 [0.90, 0.97]	<0.005

AP: antero posterior; CI: confidence interval; ICC: intraclass correlation coefficient; LA: left atrial; LAA: left atrial appendage; ML: medio lateral; PV: pulmonary vein; SD: standard deviation; SI: supero inferior.

### 3.5 Subgroup performance

Performance remained consistent across clinically relevant subgroups. For patients with versus without AF (n = 130 vs. n = 277), LA volume ICC was 0.998 (95% CI: 0.997–0.999) versus 0.999 (0.998–0.999); p = 0.412. Among patients with LA enlargement (volume >116 mL, n = 140), segmentation DSC was 0.981 (0.975–0.986) versus 0.985 (0.981–0.988) for normal LA; p = 0.228. Cases with PV anatomical variants (common trunks, n = 23) showed acceptable PV ostial ICC of 0.79 (0.66–0.87) versus 0.86 (0.82–0.90) for typical anatomy; p = 0.048, with most variants classified as Grade B.

### 3.6 Failure analysis and error modes

**FIGURE 5:**
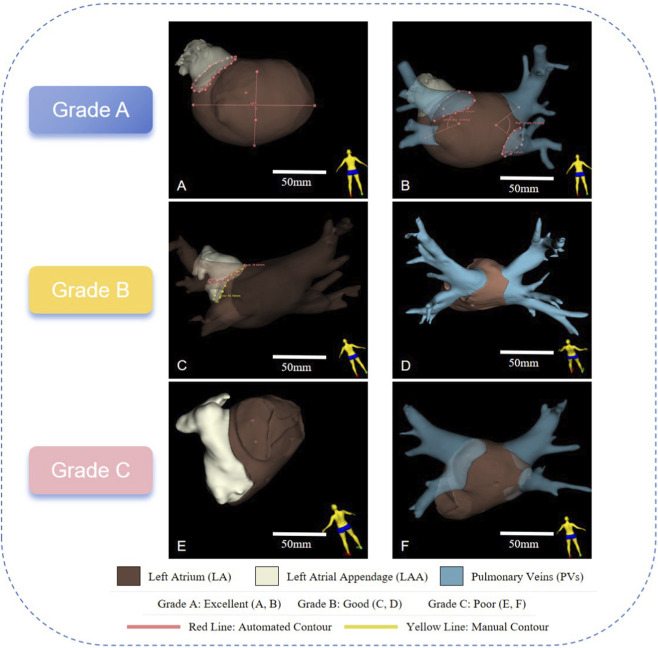
Representative examples of automated grading outcomes and failure modes. **(A, B)** Grade A: case demonstrating accurate and complete metric extraction, including consistent LA diameters, PV ostial planes, and LAA segmentation. **(C, D)** Grade B: examples requiring minor manual adjustment. Panel C illustrates a case with aberrant LAA segmentation, while Panel D shows a case with a PV common trunk resulting in abnormal ostial-plane placement. **(E, F)** Grade C: case with major errors, in which both LAA and PV segmentations were inaccurate, precluding reliable metric generation.

Among the 137 analyzed cases, 45 (32.8%) were categorized as Grade B or C, including 32 of 87 cases (36.8%) in the validation cohort and 13 of 50 cases (26.0%) in the clinical evaluation cohort. The principal sources of error were: (i) atypical pulmonary venous (PV) anatomy, such as common trunks or accessory veins, which resulted in unstable ostial-plane determination (15/45, 33.3%); (ii) suboptimal left atrial appendage (LAA) segmentation leading to inaccuracies in LAA volume or ostial-plane estimation (14/45, 31.1%); and (iii) plane-localization errors at the LA–PV junction requiring manual correction (16/45, 35.6%). Representative examples comparing automated plane placement (solid lines) with expert-adjudicated corrections (dashed lines) are shown in Figure 5.

## 4 Discussion

We developed a definition-transparent pipeline that reproduces eight clinically routine LA metrics-LA volume, LAA volume, AP/ML/SI diameters, PV ostial equivalent diameters (left/right), PV inter-ostial angles (left/right), and LAA ostial equivalent diameter—with expert-level agreement and substantial time savings. Most cases were classified as Grade A, indicating direct usability without manual edits, while Grade B highlighted limited, localized review (most often at PV ostia).

LA volume and diameters (particularly AP and SI) are routinely used to estimate AF burden and recurrence risk (Tsang et al., 2006; Beukema et al., 2005; Marchese et al., 2012). Our framework automatically quantifies these indices with excellent agreement compared to expert measurements, while reducing end-to-end measurement time by ∼92%. This combination of speed and reproducibility supports standardized pre-procedural planning and longitudinal follow-up where consistent measurements are essential (Packer et al., 2019; Marrouche et al., 2014).

Large or anatomically variant PV ostia and atypical inter-ostial angles can complicate catheter navigation, lesion formation, and durable isolation (Yamane et al., 2002; Lin et al., 2003; Pappone et al., 2003). To our knowledge, this is the first CTA-based workflow to provide fully automated, side-specific PV ostial equivalent diameters together with left/right inter-ostial angles within a unified geometric framework. Agreement and distribution analyses confirmed close consistency with manual practice, thereby enabling trajectory planning and potentially informing sheath and catheter selection (Dong et al., 2006; Kistler et al., 2006).

Accurate LAA quantification is central to device sizing in LAAC (Kar et al., 2021; Baman et al., 2018; Reddy et al., 2017). Our pipeline directly outputs LAA volume and ostial equivalent diameter from CTA with high reproducibility and minimal operator interaction. By adhering to conventional definitions and providing usability grades, it streamlines sizing workflows while flagging cases requiring verification. However, prospective validation of device sizing accuracy and procedural outcomes remains necessary before clinical deployment (Galea and Räber, 2023; Dukkipati et al., 2018).

The proposed A/B/C grading scheme offers a practical bridge from manual to automated workflows. Grade A cases can be reported without edits, Grade B prompts targeted review, and Grade C requires full manual assessment. This design promotes safe clinical adoption without pre-assuming outcome data (Cruz-González et al., 2017), while also highlighting specific targets for iterative model refinement.

Previous work has largely emphasized segmentation accuracy or outcome prediction (Zheng et al., 2018; Joyce et al., 2018; Xiong et al., 2019). Our emphasis on definition-traceable geometric metrics-computed identically from automated and manual masks-reduces ambiguity, enhances auditability, and facilitates integration into planning software (Fonseca et al., 2011; Tobo et al., 2015). This approach clarifies that residual discrepancies originate from segmentation differences rather than inconsistencies in measurement definitions.

## 5 Limitations

First, single-center data from one scanner vendor (Philips iCT 256) limits generalizability; inter-scanner differences in reconstruction and contrast protocols may affect performance (Petersen et al., 2016). A multi-center validation across Siemens, GE, and Philips scanners is underway (Isensee et al., 2021). Second, prospective clinical impact (e.g., ablation success, LAAC device sizing accuracy) has not been demonstrated. Third, the grading system reflects expert consensus rather than outcome-validated thresholds (Razeghi et al., 2020; Zuluaga et al., 2013). Fourth, edge cases (anatomical variants, motion artifacts) require expert oversight (Grade B/C) (Razeghi et al., 2020; Zuluaga et al., 2013). Finally, while adequate for ICC precision, the evaluation cohort (n = 50) may not capture all rare configurations.

Future directions include completing external validation (Isensee et al., 2021), prospective workflow assessment in electrophysiology and LAAC clinics (Peng et al., 2016), integration with electroanatomic mapping systems (CARTO, EnSite) and PACS, and extension to additional metrics (ostial eccentricity, LAA morphology) (Varela et al., 2017). These steps will establish clinical utility beyond technical feasibility (Peng et al., 2016; Ecabert et al., 2008).

## Data Availability

The original contributions presented in the study are included in the article/supplementary material, further inquiries can be directed to the corresponding author.
